# Outer Retinal and Choroidal Changes in Adolescents with Long-Lasting Type 1 Diabetes

**DOI:** 10.3390/jcm13010229

**Published:** 2023-12-30

**Authors:** Elisabetta Pilotto, Eleonora Cosmo, Tommaso Torresin, Marco Coppola, Joaquin Gutierrez De Rubalcava Doblas, Giulia Midena, Carlo Moretti, Edoardo Midena

**Affiliations:** 1 Department of Neuroscience—Ophthalmology, University of Padova, 35128 Padova, Italy; eleonora.cosmo@unipd.it (E.C.); tommaso.torresin@gmail.com (T.T.); marco.coppola@studenti.unipd.it (M.C.); edoardo.midena@unipd.it (E.M.); 2 Pediatric Diabetes Unit, Department of Women’s and Children’s Health, University of Padova, 35128 Padova, Italy; joaquin.gutierrezderubalcava@aopd.veneto.it (J.G.D.R.D.); carlo.moretti@aopd.veneto.it (C.M.); 3 IRCCS—Fondazione Bietti, 00198 Rome, Italy; giulia.midena@fondazionebietti.it

**Keywords:** type 1 diabetes, adolescents, OCT, OCT angiography, retinal pigment epithelium, outer retina, diabetic retinopathy, choroid, choriocapillaris, continuous glucose monitoring

## Abstract

This study aimed to assess outer retinal layer (ORL), retinal pigment epithelium (RPE), choroid (Ch) and choriocapillaris (CC) modifications in adolescents with long-lasting (>10 years) type 1 diabetes (T1D) without (noDR) or with diabetic retinopathy (DR). ORL and RPE thickness were measured at optical coherence tomography (OCT) macular scans. Vascular parameters of Ch and CC were quantified after elaboration of macular OCT-angiography (OCTA) images. Insulin dose and auxological and metabolic parameters were correlated with OCT and OCTA findings in patients. ORL thickness was higher in DR eyes than in noDR and healthy controls (HC), and RPE thickness was higher in noDR and DR eyes than in HC, with statistical significance for some sectors in noDR versus HC. No OCTA parameters of CC and Ch differed among groups, and no significant correlation was observed with auxological and metabolic parameters. In conclusion, ORL and RPE were both increased in adolescents with long-lasting T1D. Such changes were not associated with insulin dose and glycemia control, nor to any choroid or choriocapillaris flow change clinically detectable at OCTA, and they could be potential imaging biomarkers of disease progression.

## 1. Introduction

Type 1 diabetes (T1D) is the most frequent metabolic disease among children and adolescents, carrying several complications such as diabetic retinopathy (DR). The primary cause of diabetic retinal disease seems to be the damage of the neurovascular unit [1,2,3], which is represented by endothelial cells and pericytes, retinal neurons and glial cells, including both micro- and macroglia. Such damage induces morphological and microvascular retinal changes, detectable by means of optical coherence tomography (OCT) and OCT angiography (OCTA), respectively, even in young patients with T1D before the appearance of clinical signs of DR [4,5,6]. It has been widely demonstrated that neurodegeneration induced by diabetes starts much earlier than the microvascular changes characterizing DR [4,7,8,9]. Our group has recently reported morphological retinal changes in a cohort of adolescents with childhood-onset long-lasting T1D with or without DR, detecting an increase in volume and thickness of the ganglion cell layer and of the outer retina, this last one intended as the layers ranging from the external limiting membrane to the retinal pigment epithelium (RPE)–Bruch’s membrane complex [5]. In the present study, via a post hoc analysis of the former reported T1D adolescents cohort, we aimed to separately evaluate the contribution of the outer neuroretinal and RPE–Bruch complex layers in the total outer retina thickness increase, previously detected in T1D adolescents with or without clinical DR. Moreover, as the choroid has an important role in maintaining the metabolic demands of the outer retinal layers and retinal pigment epithelium, in the present study choroidal and choriocapillaris vascular changes were additionally evaluated with OCTA. Since all patients used continuous glucose monitoring (CGM) for the daily management of the disease, following recommendations of the Advanced Technologies and Treatments for Diabetes (ATTD) consensus, we correlated the OCT and OCTA findings with the CGM metrics, glycate hemoglobin (HbA1c) value, insulin dose and auxological parameters [10,11].

## 2. Materials and Methods

### 2.1. Study Population

This was a retrospective analysis of data tracked in a prospectively observational cross-sectional study, conducted in accordance with the tenets of the Declaration of Helsinki and approved by the Ethics Committee of the University of Padova. Informed consent was obtained from each studied subject or from a legal guardian in patients < 18 years old. The study included adolescents affected by long-lasting (>10 years) T1D with childhood-onset, followed at the Pediatric Diabetes Unit of the Padova University Hospital, who underwent OCT and OCTA (Spectralis HRA + OCT; Heidelberg Engineering, Heidelberg, Germany) and full ophthalmological examination. As previously reported [5], inclusion criteria were a diagnosis of T1D according to the World Health Organization classification [11], age ≤ 20 years old, duration of the disease > 10 years and high quality of the OCT and OCTA captured images. Exclusion criteria were a positive history for prematurity, any abnormality of the retina or choroid that could affect OCT analysis (i.e., hereditary retinal disease, vitreoretinal diseases, more than 3 diopters of myopia or hyperopia and previous uveitis), positive history for glaucoma or intraocular pressure ≥ 21 mmHg, neurodegenerative diseases or other neurological disorders independent from diabetes and low quality of the OCT captured images. Each patient underwent a complete ophthalmological evaluation including fundus ophthalmoscopy to assess the presence of clinical signs of diabetic retinopathy, which was classified according to the Clinical Diabetic Retinopathy Disease Severity Scale [12]. T1D patients were then divided into two groups, according to the presence (DR group) or absence (noDR group) of DR. Fifteen healthy adolescents served as healthy controls (HC group). 

### 2.2. Imaging 

**Figure 1:**
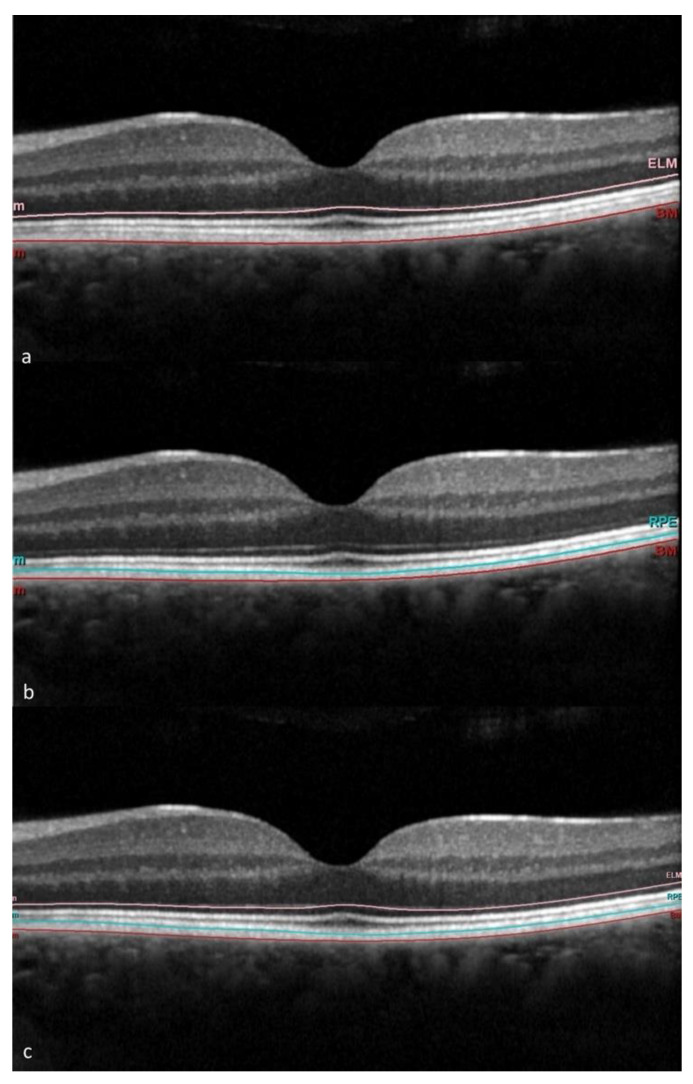
OCT scans of a noDR patient with the automatic segmentation provided by Spectralis software: (**a**) outer retinal layers, ranging from the external limiting membrane (ELM) in rose to the Bruch membrane (BM) in red; (**b**) retinal pigment epithelium (RPE) delimited by the photoreceptors separation line in light blue and BM in red; (**c**) “Outer retinal layers slab minus RPE slab” (ORL), including ELM plus myoid zone, ellipsoid zone and outer segments of the photoreceptors.

For each studied eye, an OCT 20° × 20° volumetric macular map and an OCTA 10° × 10° macular map centered onto the foveola were captured. OCT and OCTA analysis were performed by a masked operator. As regards OCT, a circular area of 6 mm in diameter centered onto the foveola and corresponding to an ETDRS grid automatically provided by the Spectralis software (Heyex version 2.5.5, Heidelberg Eye Explorer, Heidelberg Engineering, Heidelberg, Germany) was analyzed. The ETDRS grid further divided the analyzed area into the following nine zones: a central circular zone of 1 mm in diameter, an inner ring of 3 mm in diameter subdivided into four quadrants (inner superior, inner temporal, inner inferior and inner nasal) and an outer ring of 6 mm in diameter also subdivided into four quadrants (outer superior, outer temporal, outer inferior and outer nasal). Retinal layers were automatically segmented by the incorporate Spectralis software (Heyex version 2.5.5) which identified the outer retinal layers (ORL) as the layers ranging from the external limiting membrane to the RPE–Bruch complex [5]. For the present post hoc analysis, we used a further tool of the Spectralis software to subtract the RPE–Bruch complex from the total ORL, in order to obtain two distinct layers, the RPE–Bruch complex (which, from now on, we refer to as “RPE”) and the “ORL minus RPE”, including the external limiting membrane plus the myoid zone, ellipsoid zone and outer segments of the photoreceptors (which, from now on, we refer to as “ORL”) (Figure 1). The automatic retinal segmentation provided by the OCT was eventually revised manually when needed. The following measures of the ORL and RPE layers were considered: the mean thickness (global thickness) of the whole ETDRS area and the mean thickness of each of the nine ETDRS subfields. All measures were automatically provided by the incorporated software of the Spectralis device.

**Figure 2:**
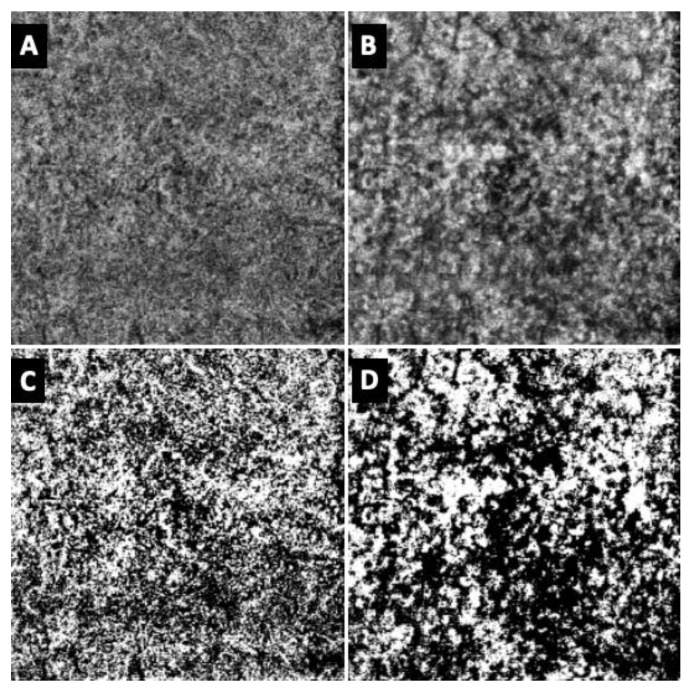
OCTA en-face images of the choriocapillaris (**A**) and of the choroid (**B**) of a healthy subject as obtained from a 10° × 10° macular map centered onto the foveola. Elaboration of the same images by means of ImageJ software (version 1.53s, National Institutes of Health, Bethesda, MD, USA) for choriocapillaris (**C**), and for choroid (**D**).

En-face OCTA images of the choriocapillaris (CC) and the choroid (Ch) were obtained from the macular map by means of the in-built segmentation algorithm, as previously described [5]. The analysis of the OCTA images was carried out by means of open-source available ImageJ software (version 1.53s, National Institutes of Health, Bethesda, MD, USA). Images were elaborated as previously described [13] and three parameters were obtained: the vessel density (VD), defined as the proportion between the luminal area represented by white pixels and the total area, the stromal density (SD), defined as 1—VD, and the vascular/stromal ratio (V/S ratio) (Figure 2).

### 2.3. Systemic Parameters 

The following standardized CGM metrics were recorded considering the average and range of variability (difference between the minimum and maximum value) of the three-monthly measures of the last year [5]: mean glucose (mg/dL), glycemic variability (standard deviation value of blood glucose: SD), time in range (% of readings within 70–180 mg/dL, TIR) and time below range (% of readings < 70 mg/dL, TBR). 

The following systemic parameters, considering the last year values, were also collected: HbA1c (%), total and pro kilo insulin dose, divided into basal and loading dose, weight, body mass index (BMI) and BMI percentile adjusted for age and gender. 

### 2.4. Statistical Analysis 

Quantitative parameters were expressed as the mean value ± standard deviation (SD), a range of minimum and maximum was also indicated; qualitative parameters were expressed as absolute and relative frequency in percentages. The normal distribution of data was analyzed by the Shapiro–Wilk test. The inferential analysis was performed firstly comparing T1D patients and HC, then comparing the three groups: DR, noDR and HC. To compare the three groups (DR, noDR and HC), a mixed-effect analysis of variance model (PROC MIXED) with repeated measurements in both eyes of the same patient was used. Two main factors (group and sector) with interaction (group × sector) were included in the model and the comparison between couples of groups in each of the 9 ETDRS sectors was performed using the Tukey–Kramer correction for multiple comparison. After this, another analysis was conducted on diabetic groups to assess the correlation between retinal parameters and insulin dose, auxological and metabolic parameters. A multiple linear regression model (adjusted for gender, age and replications of measurements considering both eyes of each patient) was estimated for each variable. The regression coefficient was tested for statistical significance and used to express the extent of the correlation and its direction (direct if the sign of the coefficient was positive, inverse if it was negative). Analyses were carried out using SAS 9.4 statistical software (SAS Institute, Cary, NC, USA) on a personal computer. Statistical tests results were considered significant if *p* < 0.05.

## 3. Results

### 3.1. Population Features

A total of 39 T1D patients and 20 HC subjects had been enrolled [5]. For the present post hoc analysis, 3 T1D patients and 5 HC were excluded due to poor OCT and/or OCTA imaging quality for the present analysis. Consequently, 36 T1D patients, 15 (41.6%) males and 21 (58.3%) females, and 15 HC subjects, 4 (26.6%) males and 11 (73%) females, were considered. In total, 5 T1D patients had ophthalmoscopic signs of DR (DR group, 10/72 eyes, 13.8%) and 31 had no clinical DR (noDR group, 62/72 eyes, 86.1%). The mean age was 17.2 ± 2.0 years in the noDR group, 17.0 ± 1.6 years in the DR group and 17.3 ± 3.1 years in the HC group, with no significant differences among groups. The mean duration of diabetes was 10.6 ± 4.07 years in the DR group and 13.0 ± 2.80 years in the noDR group, with no significant difference. Data are reported in Table 1.

The CGM indices, insulin dose, auxological and metabolic parameters of DM1 patients are reported in Table 2; there were no significant differences among the DR and noDR groups.

### 3.2. OCT and OCTA Parameters

The global and sectorial ORL thickness were thicker in DR eyes than in noDR and controls’ eyes, reaching statistical significance for some sectors. We did not find any difference among the noDR and HC groups (Table 3).

Global and sectorial RPE thickness were thicker in diabetic eyes (both in noDR and DR eyes) than in HC eyes, reaching statistical significance for some sectors between the noDR and HC groups. No differences were found between the noDR and DR groups. (Table 4).

No significant correlation was observed between the ORL thickness and GCM indices, insulin dose, auxological and metabolic parameters. In the noDR group, RPE thickness directly correlated with BMI (*p* = 0.01). No other significant correlations were observed among the other analyzed parameters. 

None of the OCTA parameters (VD, SD and V/S ratio) of CC and Ch significantly differed among groups. The results are reported in Table 5.

## 4. Discussion

In this post hoc analysis, we investigated ORL and RPE, analyzing them separately, to understand which of them was responsible of the previously detected ORL–RPE complex increase in adolescents with long-lasting T1D. We detected that both layers were increased, with some differences according to the absence or presence of clinical DR. 

The increase in the RPE layer in T1D patients was present in both noDR and DR patients, significantly for some sectors in noDR compared to controls. It has been demonstrated in T1D animal models that hyperglycemia induces an early osmotic increase in RPE cellular volumes [14]. Early RPE cell changes have also been detected by in vitro studies and proteomic analysis of RPE cells of diabetic human donor eyes with no clinical DR [15,16,17,18,19,20]. In T1D adolescents, the long-lasting hyperglycemia may induce an increase in the RPE layer thickness at the OCT, secondary to the increase in the RPE cell volume, as detected in basic research studies. The loss of significance for DR patients compared to controls, in our results, could be related to their limited number. A long follow-up study including a larger population of patients with clinical signs of DR might highlight eventual differences between patients with and without DR.

Our study showed that the ORL layer was also increased in T1D adolescents. To date, neurodegeneration in diabetes-related damage even in preclinical stages is well established, as abnormalities of the neural retina, mainly involving ganglion cells, Müller cells, horizontal cells, astrocytes and microglia, have been reported in experimental and human diabetes [4,8,21,22,23,24,25,26,27]. On the contrary, the effect of diabetes on photoreceptors is still under debate, since until now inconsistent conclusions have been reached from both human and animal studies. Moreover, such studies have mainly investigated the outer nuclear layer (ONL), containing only the nuclei of photoreceptors [18,28]. In the present study, the outer retinal layers, corresponding to the inner and outer segments of photoreceptors, have been analyzed, as the ONL did not differ between T1D adolescents and healthy controls in our previous analysis [5]. In addition, to date no studies have investigated ORL changes at OCT in T1D children or adolescents; thus, our study is the first to assess such alterations in a population of long-lasting-T1D adolescents. In adults with T1D, with or without clinical signs of DR, no significant ORL changes in volume and/or thickness have been reported [29,30]. However, Pemp et al. reported, despite a mild and not significant decrease in the volume of many retinal layers such as the ganglion cell, inner plexiform, inner nuclear, outer plexiform and outer nuclear layer, that the photoreceptor layer volume was the only one mildly increased in T1D adults with mild–moderate DR compared to healthy controls [30].

Degenerative changes of photoreceptors, with swelling of inner segments, containing rounded and swollen mitochondria and vacuoles of various sizes, had been detected in streptozotocin-induced diabetes, without the detectable loss of photoreceptors [14]. Such findings have also been confirmed in in vivo studies conducted in T1D adolescents, using adaptive optics retinal imaging [31]. The results from our study seem to confirm that, in young patients with T1D, structural changes happen in retinal photoreceptors, with an increase in their inner and outer segments, which might perhaps precede the cell loss detectable in older patients [32,33]. The evaluation of photoreceptors’ function was beyond the aim of our study; therefore, whether these changes have a functional counterpart cannot be elucidated. Our results showed that the increase in ORL thickness mainly affected DR eyes rather than noDR eyes. The more evident increase in ORL, probably corresponding to the aforementioned swelling of photoreceptors’ mitochondria with increased in oxygen consumption and demand, in DR compared to noDR eyes, could corroborate the hypothesis that photoreceptors might influence the development of overt DR [34,35].

Since the outer retina is primarily dependent on diffusion from choroidal circulation for its oxygen and nutrients demand, we may expect an association between photoreceptors’ alterations and choriocapillaris and choroid changes. Histopathological studies have detected the loss of choriocapillaris in human diabetic eyes [36,37], and with the advent of OCTA, an increasing number of studies have recently highlighted a reduced choriocapillaris blood flow in vivo, in adults with T1D and T2D, worsening with the DR severity increase [33,37,38,39,40,41,42]. Conversely, in the present study, no choroidal or choriocapillaris flow changes were detected in T1D adolescents, with or without DR, compared to controls. This is in line with previous studies conducted on a T1D child population that reported no changes in choroidal thickness in OCT [43,44]. We believe that such findings may be due to the demographic features of our cohort, in particular the patients’ age. Indeed, it has been demonstrated that when puberty occurs choroidal thickness increases because of the change in hormone levels, both in T1D and in healthy adolescents [45,46,47]. This might suggest that T1D does not affect the choroid and choriocapillaris during adolescence, and therefore, that the detected ORL changes are not related to the choroidal circulation. In fact, apart from the choroidal circulation, photoreceptors partly rely on the retinal circulation for their oxygen needs, mainly in eyes with DR [48,49].

One of the main limits of the present study was the small size of the analyzed cohort. On the other side, its strength was the enrollment of a population of adolescents homogeneous for age, disease duration and treatment regimen, deriving from a single tertiary pediatric diabetes unit. Another limit was the small number of patients with DR. In this cohort of T1D adolescents with early-onset and long-lasting disease, the prevalence of DR was 14%, lower than expected considering the long duration of the disease and lower compared to that of an adult T1D population (70% in Viggiano’s study) [33], but superior to that of a previously reported young T1D population (3% in Mameli’s study) [6]. The present study included young patients, consecutively enrolled, affected by early-onset long-lasting T1D with or without clinical DR, and despite the different distribution that might have affected the loss of some significance, some significant differences were detected between noDR and DR patients. 

## 5. Conclusions

In conclusion, we detected that the outer neuroretina and RPE layers are both increased in T1D adolescents. These layers changes are not associated with any choroid or choriocapillaris flow changes clinically detectable in OCTA. Follow-up studies in young diabetic populations would be useful to better understand the pathophysiology of the external retinal layers changes thus far reported and their eventual functional and retinal vascular counterpart.

## Figures and Tables

**Table 1:** Study population demographic parameters.

Parameter	DR Group (N = 5)	noDR Group (N = 31)	HC Group (N = 15)
Sex (males:females)	2:3	13:18	4:11
Mean age ± SD (years)	17.0 ± 1.6	17.2 ± 2.0	17.3 ± 3.1
T1D mean duration ± SD (years)	10.6 ± 4.07	13.0 ± 2.80	n.a.

DR: diabetic retinopathy; HC: healthy control; N: number; SD: standard deviation; T1D: type 1 diabetes; n.a.: not applicable.

**Table 2:** Continuous glucose monitoring (CGM) indices, insulin dose, auxological and metabolic parameters of T1D patients.

Parameter	DR Group	noDR Group	*p*-Value
Mean glucose ± SD (mg/dL)	179.6 ± 14.0	178.2 ± 30.9	0.864
Glycemic variability (SD) ± SD (mg/dL)	84.4 ± 7.0	80.0 ± 14.7	0.339
TIR ± SD (%)	48.3 ± 3.9	46.6 ± 11.2	0.549
TBR ± SD (%)	9.3 ± 4.7	9.7 ± 6.9	0.880
HbA1c ± SD (%)	7.7 ± 0.9	7.6 ± 1.0	0.773
Weight (kg)	64.4 ± 10.3	62.9 ± 11.3	0.783
BMI (kg/m^2^)	23.7 ± 3.1	21.2 ± 2.57	0.066
Total insulin daily dose (IU/die)	61.8 ± 28.0	53.2 ± 14.5	0.296
Insulin daily dose adjusted for weight (IU/kg/die)	0.95 ± 0.3	0.85 ± 0.21	0.377
Basal insulin over total (%)	47.4 ± 16.3	54.8 ± 12.7	0.251
Insulin bolus over total (%)	52.6 ± 16.3	45.1 ± 12.7	0.251

DR: diabetic retinopathy; SD: standard deviation; TIR: time in range; TBR: time below range; HbA1c: glycate hemoglobin; BMI: body mass index; IU: international units.

**Table 3:** Outer retinal layer (ORL) thickness (global and ETDRS sectors), expressed in µm.

Sector	HC Group Mean ± SD (*n* = 30 Eyes)	DR Group Mean ± SD (*n* = 10 Eyes)	noDR Group Mean ± SD (*n* = 62 Eyes)	*p*-Value DR vs. noDR	*p*-Value DR vs. HC	*p*-Value noDR vs. HC
Global	67.2 ± 1.7	68.4 ± 1.9	67.6 ± 1.9	0.241	0.202	0.492
Central	74.7 ± 3.8	75.6 ± 5.1	75.8 ± 3.7	0.631	0.481	0.113
IS	66.7 ± 2.0	67.8 ± 1.8	67.1 ± 2.2	0.439	0.374	0.612
IN	68.5 ± 2.6	68.7 ± 1.4	68.8 ± 2.6	0.761	0.956	0.707
II	66.5 ± 1.9	67.1 ± 2.0	66.8 ± 3.2	0.784	0.670	0.742
IT	68.2 ± 2.4	68.6 ± 1.6	68.4 ± 2.5	0.902	0.762	0.764
OS	65.5 ± 2.2	67.1 ± 2.4	65.9 ± 2.0	0.159	0.158	0.540
ON	65.5 ± 2.0	67.5 ± 3.4	66.0 ± 2.2	0.078	0.045 *	0.437
OI	63.8 ± 2.3	66.0 ± 3.4	64.3 ± 2.1	0.040 *	0.045 *	0.535
OT	65.2 ± 2.0	67.1 ± 3.2	65.3 ± 2.2	0.030 *	0.104	0.969

DR: diabetic retinopathy; HC: healthy control; SD: standard deviation; IS: inner superior; IN: inner nasal; II: inner inferior; IT: inner temporal; OS: outer superior; ON: outer nasal, OI: outer inferior; OT: outer temporal. * Statistically significant values in bold.

**Table 4:** Retinal pigment epithelium (RPE) thickness (global and ETDRS sectors), expressed in µm.

Sector	HC Group Mean ± SD (*n* = 30 Eyes)	DR Group Mean ± SD (*n* = 10 Eyes)	noDR Group Mean ± SD (*n* = 62 Eyes)	*p*-Value DR vs. noDR	*p*-Value DR vs. HC	*p*-Value noDR vs. HC
Global	14.8 ± 1.1	15.6 ± 1.7	15.5 ± 1.1	0.967	0.353	0.083
Central	18.5 ± 1.7	18.5 ± 2.3	18.5 ± 1.8	0.152	0.350	0.960
IS	14.9 ± 1.4	15.6 ± 1.8	15.8 ± 1.4	0.419	0.471	**0.038** *
IN	15.8 ± 1.6	16.6 ± 1.8	16.4 ± 1.3	0.937	0.391	0.172
II	14.7 ± 1.5	15.7 ± 2.2	15.4 ± 2.3	0.684	0.239	0.118
IT	14.3 ± 1.8	15.5 ± 2.1	15.4 ± 1.5	0.943	0.159	**0.014** *
OS	14.0 ± 1.3	14.9 ± 1.4	14.6 ± 1.4	0.700	0.277	0.153
ON	14.7 ± 1.4	15.1 ± 2.0	14.9 ± 1.4	0.824	0.690	0.665
OI	13.6 ± 1.1	14.6 ± 1.5	14.5 ± 1.4	0.864	0.221	**0.024** *
OT	12.8 ± 1.6	14.0 ± 1.9	13.5 ± 1.5	0.357	0.146	0.139

DR: diabetic retinopathy; HC: healthy control; SD: standard deviation; IS: inner superior; IN: inner nasal; II: inner inferior; IT: inner temporal; OS: outer superior; ON: outer nasal, OI: outer inferior; OT: outer temporal. * Statistically significant values in bold.

**Table 5:** OCTA parameters of the choriocapillaris and choroid.

Parameter	HC Group Mean ± SD (*n* = 30 Eyes)	T1D Group Mean ± SD (*n* = 72 Eyes)	DR Group Mean ± SD (*n* = 10 Eyes)	noDR Group Mean ± SD (*n* = 62 Eyes)	*p*-Value T1D vs. HC	*p*-Value DR vs. noDR	*p*-Value DR vs. HC	*p*-Value noDR vs. HC
CC VD	0.434 ± 0.038	0.423 ± 0.035	0.418 ± 0.031	0.424 ± 0.036	0.2794	0.577	0.185	0.219
CC SD	0.566 ± 0.038	0.577 ± 0.035	0.582 ± 0.031	0.576 ± 0.036	0.2794	0.577	0.185	0.219
CC V/S ratio	0.775 ± 0.115	0.741 ± 0.107	0.723 ± 0.093	0.744 ± 0.110	0.2617	0.537	0.159	0.202
Ch VD	0.376 ± 0.052	0.374 ± 0.056	0.398 ± 0.047	0.365 ± 0.054	0.9266	0.067	0.208	0.380
Ch SD	0.624 ± 0.052	0.626 ± 0.056	0.602 ± 0.047	0.635 ± 0.054	0.9266	0.067	0.208	0.380
Ch V/S ratio	0.612 ± 0.138	0.610 ± 0.141	0.671 ± 0.132	0.587 ± 0.129	0.9498	0.084	0.239	0.375

T1D: type 1 diabetes; DR: diabetic retinopathy; HC: healthy control; SD: standard deviation; CC: choriocapillaris; Ch: choroid; VD: vascular density; SD: stromal density; V/S ratio: vascular/stromal ratio.

## Data Availability

The data presented in this study are available in the article. Eventual additional data are available on request from the corresponding author.
